# Supplementary material for: Whole-brain annotation and multi-connectome cell typing of Drosophila
Source: Nature. 2024 Oct 2;634(8032):139–52. doi: 10.1038/s41586-024-07686-5 (PMC11446831; doi:10.1038/s41586-024-07686-5)
Supplement: Supplementary file 1 — Supplementary Tables 1–3 and detailed explanations of the columns in Supplementary Data 1–5. [file 41586_2024_7686_MOESM1_ESM.pdf]

nature portfolio

https://doi.org/10.1038/s41586-024-07686-5

Supplementary information

Whole-brain annotation and multi-connectome cell typing of *Drosophila*

In the format provided by the authors and unedited

nature portfolio

https://doi.org/10.1038/s41586-024-07686-5

Supplementary information

Whole-brain annotation and multi-connectome cell typing of *Drosophila*

In the format provided by the authors and unedited

Supplementary Tables

flow	superclass	class	sub class	type	side	hemilineage	neurotransmitter
afferent	sensory	olfactory	ALRN	ORN_DA1	left	ALl1_ventral	acetylcholine
intrinsic	central	DAN		PAM08	left	CREa1_dorsal	dopamine
efferent	descending			DNa02	right	WEDd1	acetylcholine

Supplementary Table 1: Sets of annotations for three example neurons.

Field	Value	Ontology ID	Definition
flow	intrinsic		Neurons fully contained within the brain.
	afferent		Neurons that enter the brain from the periphery or the ventral nerve cord (VNC).
	efferent		Neurons that leave the brain towards the periphery or the VNC.
superclass	central	FBbt_00059245	Neurons fully contained within the central brain.
	ascending	FBbt_00048301	Neurons entering the brain from the VNC. These can be sensory or interneurons.
	descending	FBbt_00047511	Neurons with soma in the brain that exit the brain towards the VNC.
	endocrine	FBbt_00059246	Neurons that exit the brain via the NCC towards the retrocerebral complex (corpora cardiaca and corpora allata).
	motor	FBbt_00005123	Neurons that exit the brain towards the periphery (and are hence assumed to be motor neurons).
	optic	FBbt_00007577	Neurons fully contained within the optic lobes or the ocellar ganglion. Includes some bilateral neurons (see class field).
	visual projection	FBbt_00048287	Neurons that have dendrites in the optic lobes or the ocellar ganglion and axons in the central brain.
	visual centrifugal	FBbt_00059244	Neurons that have dendrites in the central brain and axons in the optic lobes or the ocellar ganglion.
	sensory	FBbt_00005124	Neurons that enter the brain from the periphery. Note that “ascending” also includes some sensory neurons that we are unable to distinguish from ascending interneurons with any certainty.

Supplementary Table 2: Glossary for terms used in the top-most layers of the annotation hierarchy. Ontology ID refers to the Virtual Fly Brain database.

**Class**	**Subset of superclass**	**Definition**
bilateral	optic	Optic lobe neurons with projections into the contralateral optic lobes. Sometimes also leave synapses in the central brain.
ocellar	visual centrifugal	Projection neurons with dendrites in the brain and axons in the ocellar ganglia.
	visual projection	Projection neurons with dendrites in the ocellar ganglia and axons in the brain.
	optic	Neurons intrinsic to the ocellar ganglia. Cell bodies can be inside the brain though.
ALIN	central	“Antennal lobe input neurons”: neurons with dendrites out- and axons inside the antennal lobes. Does not include sensory neurons.
ALPN	central	“Antennal lobe projection neurons”: neurons with dendrites in the antennal lobe and axonal projections into the protocerebrum, lateral horn or calyx.
ALON	central	“Antennal lobe output neurons”: neurons with dendrites in the antennal lobes and axonal projections outside of the brain that are not canonical ALPNs.
ALLN	central	“Antennal lobe local neurons”: neurons with both dendrites and axons contained to the antennal lobes. Can be bilateral.
CX	central	Central complex neurons as defined by Hulse *et al*.(Hulse et al. 2021)
DAN	central	Dopaminergic neurons (PAM and PPL) whose axons target the mushroom body lobes.
Kenyon Cell	central	Neurons with dendrites in the mushroom body calyx whose axons form the parallel fibre system of the mushroom body lobes.
MBIN	central	“Mushroom body input neuron”: APL or DPM.
LHCENT	central	“Lateral horn centrifugal neurons”: neurons with dendrites in the protocerebrum and axons in the lateral horn.
LHLN	central	“Lateral horn local neurons”: neurons with both dendrites and axons contained to the lateral horn.
ME	optic	medulla local neuron
ME>LO	optic	projection neuron with dendrites in medulla and axon in the lobula

Supplementary Table 3: Selection of cell *classes*.

Supplementary notes

This section provides detailed explanations for columns in the Supplemental Files.

Supplemental_file1_neuron_annotations.tsv and *Supplemental_file2_non_neuron_annotations.tsv*

- *pos_x*, *pos_y*, *pos_z* are anchor coordinates in 4x4x40nm voxel space (typically on the backbone of the neuron) for given neuron
- *supervoxel_id* is the ID of the supervoxel the anchor coordinates map to
- *root_id* is the ID of the neuron in the FlyWire 783 release
- *soma_x*, *soma_y*, *soma_z* are soma coordinates in 4x4x40nm voxel space
- *nucleus_id* maps to the nucleus detection table (available through CAVE); neurons with *soma_x/y/z* but without nucleus_id have had their soma manually marked
- *flow, super_class, cell_class, cell_sub_class, cell_type, hemibrain_type* are the hierarchical annotations
- *ito_lee_hemilineage* and *hartenstein_hemilineage* provide the hemilineage in ItoLee and Hartenstein nomenclature; note that not all labels exist in the Hartenstein nomenclature
- *morphology_group* provides a coarse morphological grouping based on hemilineage clustering
- *top_nt* is the top predicted neurotransmitter for the given neuron, calculated by averaging confidences over the transmitter predictions for all presynapses for the given neuron and choosing the most confident transmitter
- *top_nt_conf* is the average confidence for the top neurotransmitter
- *side* refers to the soma side for brain-intrinsic neurons and the nerve-entry side for sensory/ascending neurons
- *fbbt_id* and *vfb_id* provide mappings to the database of the VirtualFlyBrain; the former is used for ontology terms (like cell types), the latter is an identifier for individual neurons
- *status* records a number of outliers: *outlier_seg* are neurons with segmentation issues (often due to dark cytosol); *outlier_bio* are neurons with small to medium sized differences (e.g. extra branches) compared with their contralateral or hemibrain homologues
- *known_nt* and *known_nt_source* provide information about whether there is information about a confirmed neurotransmitter for a given neuron/neuron type

Supplemental_file3_summary_with_ngl_links.csv

- *ito_lee_hemilineage* and *hartenstein_hemilineage* provide the hemilineage in ItoLee and Hartenstein nomenclature; note that not all labels exist in the Hartenstein nomenclature.
- *notes* records some of our observations of select published clones.
- *ito_lee_lineage* and *hartenstein_lineage* provide the corresponding lineage names to the hemilineages.
- *hemibrain_map* contains the *rough* mapping of that hemilineage to a hemibrain cell body fiber.

- *is_hemilineage* contains our best guesses on whether the entry is a hemilineage or not. For instance, H(NT) means that we guess that this is a hemilineage (instead of a combination of two hemilineages of one lineage) based on the neurotransmitter information from Eckstein et al. (2023).
- *ngl_link* (``, *left_*, `*right_*` and `*hemibrain_*`) columns contain neuroglancer links with the neurons in that hemilineage selected, on that side, and coloured based on the morphological groups. Each morphological group is in a separate layer (in addition, FlyWire and hemibrain neurons are in separate layers). There is also a de-selected layer that contains all neurons in that hemilineage in FlyWire.
- *ids_** (*left*, *right*, *center*, *hb*) columns contain the neuron ids for that hemilineage in that side.
- *id_count_** (*left*, *right*, *center*, *hb*) columns contain the neuron count for that hemilineage in that side.
- *n_clusters_** (*left*, *right*, *hb*) columns contain the number of clusters for that hemilineage in that side.
- *shape_truncated* and *number_truncated* columns contain information on whether the hemilineage is truncated in shape/number in the hemibrain.

Supplemental_file4_hemilineages_clustering.csv

- *root_id* is the ID of the neuron in the FlyWire 783 release.
- *persistent_cluster* column contains the side_cluster labels of neurons that cluster together across one-, two- and three-hemisphere clustering (see the Morphological groups section in Methods).
- *side* is the hemisphere (left/right/hemibrain) the neuron is in.
- *ito_lee_hemilineage* and *hartenstein_hemilineage* provide the hemilineage in ItoLee and Hartenstein nomenclature; note that not all labels exist in the Hartenstein nomenclature.
- *hemilineage_group* records the morphological groups.
- *supervoxel_id* corresponds to the supervoxel_ids in the *Supplemental_file1_neuron_annotations.tsv* file. The value is NA if the neuron is from the hemibrain.
- *nps* column contains the top-three innervated neuropils, alphabetically sorted, for that neuron.

Supplemental_file5_hemibrain_meta.csv

The following columns correspond 1:1 to columns shown/available for download through neuPrint: *bodyId, type, notes, status, cellBodyFiber, somaLocation, pre, post* and *cropped*.

We additionally added the following columns:

- in *morphology_type* we collapsed connectivity types back into morphology types (i.e. removed the _a, _b, etc. suffixes)

- *cell_class* contains labels analogous to those the cell class labels we provide for FlyWire neurons
- *side* refers to the soma side and is principally based on the instance column where an _L and _R typically indicates the side; we did however make a sizeable number of manual adjustments
- *pre_con2* contains the number of outgoing connections for a given neuron whereas pre contains the number of presynapses (remember that insect synapses are polyadic); these numbers were used to compare hemibrain vs FlyWire presynapse counts
- *fbbt_id* contains a FBbt ID that maps to the entry for a given hemibrain type in the VirtualFlyBrain database
- *ito_lee_hemilineage* provide identified hemilineages in ItoLee nomenclature
- *morphology_group* provides a coarse morphological grouping based on hemilineage clustering
