# Supplementary material for: Whole-brain annotation and multi-connectome cell typing of Drosophila
Source: Nature. 2024 Oct 2;634(8032):139–52. doi: 10.1038/s41586-024-07686-5 (PMC11446831; doi:10.1038/s41586-024-07686-5)
Supplement: Supplementary file 2 — Reporting Summary [file 41586_2024_7686_MOESM2_ESM.pdf]

nature portfolio

Corresponding author(s): Gregory Jefferis

Last updated by author(s): Mar 27, 2024

Reporting Summary

Nature Portfolio wishes to improve the reproducibility of the work that we publish. This form provides structure for consistency and transparency in reporting. For further information on Nature Portfolio policies, see our Editorial Policies and the Editorial Policy Checklist.

Statistics

For all statistical analyses, confirm that the following items are present in the figure legend, table legend, main text, or Methods section.

n/a	Confirmed	
☐	☒	The exact sample size (*n*) for each experimental group/condition, given as a discrete number and unit of measurement
☒	☐	A statement on whether measurements were taken from distinct samples or whether the same sample was measured repeatedly
☐	☒	The statistical test(s) used AND whether they are one- or two-sided<br>*Only common tests should be described solely by name; describe more complex techniques in the Methods section.*
☐	☒	A description of all covariates tested
☒	☐	A description of any assumptions or corrections, such as tests of normality and adjustment for multiple comparisons
☐	☒	A full description of the statistical parameters including central tendency (e.g. means) or other basic estimates (e.g. regression coefficient) AND variation (e.g. standard deviation) or associated estimates of uncertainty (e.g. confidence intervals)
☐	☒	For null hypothesis testing, the test statistic (e.g. *F*, *t*, *r*) with confidence intervals, effect sizes, degrees of freedom and *P* value noted<br>*Give P values as exact values whenever suitable.*
☒	☐	For Bayesian analysis, information on the choice of priors and Markov chain Monte Carlo settings
☒	☐	For hierarchical and complex designs, identification of the appropriate level for tests and full reporting of outcomes
☐	☒	Estimates of effect sizes (e.g. Cohen's *d*, Pearson's *r*), indicating how they were calculated

Our web collection on statistics for biologists contains articles on many of the points above.

Software and code

Policy information about availability of computer code

Data collection	Data collection is described in our companion paper by Dorkenwald et al. and is cited at appropriate locations throughout our manuscript.
Data analysis	For analysis we developed open-source software packages.<br>These tools are detailed in the Methods, which also includes download locations, all of which are on Github.<br>The key software packages are:<br>- navis: https://github.com/navis-org/navis v1.5.0<br>- fafbseg-py: https://github.com/navis-org/fafbseg-py v3.0.5<br>- flybrains: https://github.com/navis-org/navis-flybrains v0.2.9<br>- skeletor: https://github.com/navis-org/skeletor v1.2.3<br>- fafbseg: https://github.com/natverse/fafbseg v0.14.0<br>- coconatfly: https://github.com/natverse/coconatfly v0.1.0

For manuscripts utilizing custom algorithms or software that are central to the research but not yet described in published literature, software must be made available to editors and reviewers. We strongly encourage code deposition in a community repository (e.g. GitHub). See the Nature Portfolio guidelines for submitting code & software for further information.

Data

Policy information about availability of data

All manuscripts must include a data availability statement. This statement should provide the following information, where applicable:

- Accession codes, unique identifiers, or web links for publicly available datasets
- A description of any restrictions on data availability
- For clinical datasets or third party data, please ensure that the statement adheres to our policy

Data artefacts from this paper are available at https://github.com/flyconnectome/flywire_annotations.

This includes:
- neuron annotations + other metadata
- high quality skeletons for all proofread FlyWire neurons
- NBLAST scores for FlyWire vs. hemibrain
- all-by-all NBLAST scores for FlyWire
The repository may periodically be updated to improve annotations but older versions will always remain available via Github's versioning system.

In addition, neuron annotations + other meta data are also available for download in the supplementary materials; NBLAST scores and skeletons have been deposited in a Zenodo repository: https://zenodo.org/records/10877326 (doi: 10.5281/zenodo.10877326).

We provide a neuroglancer scene preconfigured for display and query of our annotations alongside the FlyWire neuron meshes and segmentation at http://tinyurl.com/flywire783. Users can add the annotations to arbitrary neuroglancer scenes themselves by adding a data subsource (see Extended Data Figure 11). There are two options:
"precomputed://https://flyem.mrc-lmb.cam.ac.uk/flyconnectome/ann/flytable-info-783" containing super class, cell type and side labels
"precomputed://https://flyem.mrc-lmb.cam.ac.uk/flyconnectome/ann/flytable-info-783-all" additionally contains hemi-lineage information

We also provide programmatic access to the annotations through our fafbseg R and Python packages (see Table 1 and the online documentation for examples).

Annotations have also been shared with Codex (https://codex.flywire.ai/), the connectome annotation versioning engine (CAVE) which can be queried through e.g. the CAVEclient (https://github.com/seung-lab/CAVEclient), and the FAFB-FlyWire CATMAID spaces (https://fafb-flywire.catmaid.org). At the time of writing access to Codex and CAVE requires signing up using a Google account.

To aid a number of analyses, hemibrain neuron meshes were mapped into FlyWire (FAFB14.1) space. These can be co-visualised with FlyWire neurons within neuroglancer (e.g. https://tinyurl.com/flywire783; this scene also includes a second copy of the hemibrain data (layer hemibrain_meshes_mirr) which has been non-rigidly mapped onto the opposite side of FAFB).

Research involving human participants, their data, or biological material

Policy information about studies with human participants or human data. See also policy information about sex, gender (identity/presentation), and sexual orientation and race, ethnicity and racism.

Reporting on sex and gender	Does not apply.
Reporting on race, ethnicity, or other socially relevant groupings	Does not apply.
Population characteristics	Does not apply.
Recruitment	Does not apply.
Ethics oversight	Does not apply.

Note that full information on the approval of the study protocol must also be provided in the manuscript.

Field-specific reporting

Please select the one below that is the best fit for your research. If you are not sure, read the appropriate sections before making your selection.

☒ Life sciences ☐ Behavioural & social sciences ☐ Ecological, evolutionary & environmental sciences

For a reference copy of the document with all sections, see nature.com/documents/nr-reporting-summary-flat.pdf

Life sciences study design

All studies must disclose on these points even when the disclosure is negative.

Sample size	This study analyses and compares the connectomes of two Drosophila brains.

Data exclusions	No neurons were excluded from overall annotation or analyses. For the analysis of across brain stereotypy we used a subset of the available matches; the specific exclusion criteria and rationale are clearly detailed in the methods.
Replication	Does not apply.
Randomization	Does not apply.
Blinding	Does not apply.

Reporting for specific materials, systems and methods

We require information from authors about some types of materials, experimental systems and methods used in many studies. Here, indicate whether each material, system or method listed is relevant to your study. If you are not sure if a list item applies to your research, read the appropriate section before selecting a response.

Materials & experimental systems

n/a	Involved in the study
☒	☐ Antibodies
☒	☐ Eukaryotic cell lines
☒	☐ Palaeontology and archaeology
☒	☐ Animals and other organisms
☒	☐ Clinical data
☒	☐ Dual use research of concern
☒	☐ Plants

Methods

n/a	Involved in the study
☒	☐ ChIP-seq
☒	☐ Flow cytometry
☒	☐ MRI-based neuroimaging

Plants

Seed stocks	Does not apply.
Novel plant genotypes	Does not apply.
Authentication	Does not apply.
